# APE1 mediates chemoresistance in esophageal squamous cell carcinoma by remodeling the immunosuppressive microenvironment

**DOI:** 10.3389/fimmu.2025.1689468

**Published:** 2025-10-29

**Authors:** Han Gao, Junji Rao, He Xiao, Xunjie Kuang, Yong Wang, Yujing Yao, Yuxin Yang, Mengxia Li, Xiuyong Liao, Dong Wang

**Affiliations:** ^1^ Oncology Department of Qianjiang Central Hospital of Chongqing University, School of Life Sciences of Chongqing University, School of Medicine of Chongqing University, Chongqing University, Chongqing, China; ^2^ Department of Cancer Center, Army Medical Center, Army Medical University, Chongqing, China

**Keywords:** APE1, esophageal squamous cell carcinoma, chemotherapy, survival analysis, CAF

## Abstract

**Introduction:**

Squamous cell carcinoma of the esophagus (ESCC) has poor prognosis after surgery and adjuvant chemotherapy. Biomarkers predicting treatment efficacy are urgently needed. This study investigated apurinic/apyrimidinic endonuclease 1 (APE1), a key DNA repair enzyme, as a prognostic biomarker in ESCC patients receiving postoperative chemotherapy.

**Methods:**

To assess the relationship between APE1 expression and survival outcomes post-adjuvant chemotherapy. 115 ESCC patients receiving surgery and platinum-based chemotherapy were retrospectively enrolled. APE1 expression (low, medium, high) was determined by immunohistochemistry (IHC). Furthermore, external validation was performed using a tissue microarray cohort of 110 post-chemotherapy ESCC patients and the GES5325 dataset. Survival was analyzed using Kaplan-Meier and Cox regression. The tumor immune microenvironment was characterized by multiplex immunofluorescence (mIF).

**Results:**

High APE1 expression correlated significantly with advanced T stage (*p*=0.005) and neural invasion (*p*=0.036). The high-expression group had significantly worse 5-year OS (27% vs. 91.4%) and DFS (14.3% vs. 55.3%) than the low-expression group (*p*<0.001), confirmed in public databases. Multivariate analysis identified APE1 expression (DFS: HR=4.600, 95% CI 1.285-16.466; OS: HR=16.001, 95% CI 4.826-53.061) and clinical stage as independent prognostic factors. Additionally, external validation was carried out using tissue microarrays and the GEO database to confirm the reliability. mIF analysis revealed significantly increased infiltration of FOXP3^+^ regulatory T cells (Treg) and cancer-associated fibroblasts (CAFs) in the APE1-high group.

**Discussion:**

High APE1 expression is an independent predictor of poor prognosis in ESCC patients receiving postoperative chemotherapy, associated with Treg and CAFs-mediated immunosuppression. APE1 serves as a prognostic biomarker linked to immunosuppression, enabling personalized adjuvant therapy.

## Graphical Abstract

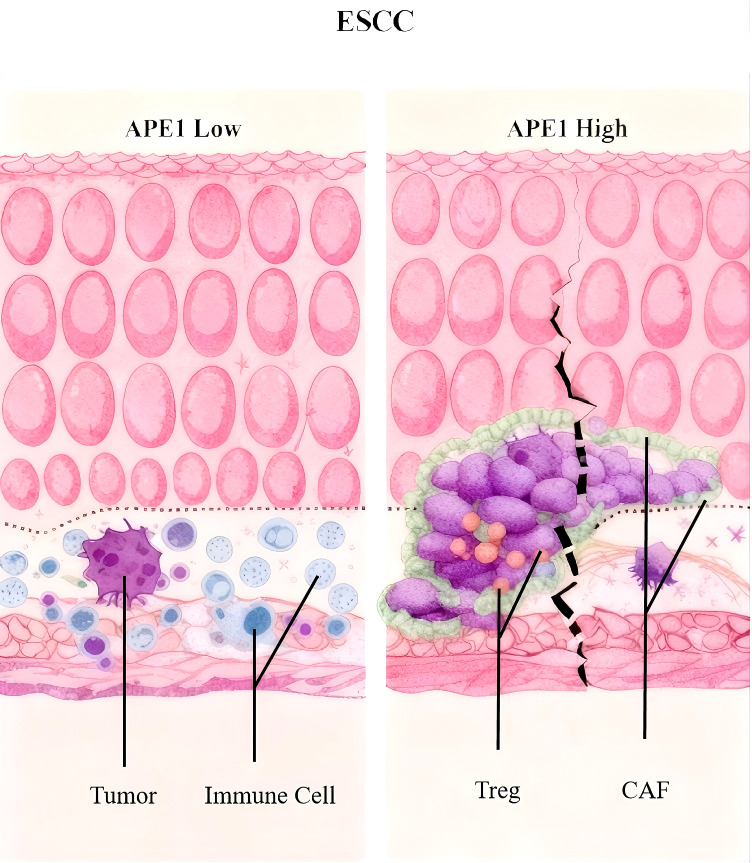

## Introduction

Esophageal cancer represents a critical global health challenge, the GLOBOCAN 2020 statistics, published by the International Agency for Research on Cancer (IARC), report that there are approximately 604,000 new cases and 544,000 deaths from esophageal cancer each year, placing it seventh in the incidence rate and sixth in the mortality rate among malignant tumors worldwide (1). Notably, China shoulders over half of the global disease burden, with its annual new cases (53.7%) and deaths (55.3%) exceeding those in other regions significantly. The age-standardized incidence rate (ASR) in China reaches 13.2 per 100,000, demonstrating a distinct geographic clustering pattern. Esophageal squamous cell carcinoma (ESCC) predominates in Asian populations, whereas esophageal adenocarcinoma (EAC) is more common in Western populations (2). Research on the present state and molecular mechanisms of esophageal cancer prevention and treatment holds substantial clinical relevance, given its status as a globally prevalent malignant tumor.

Despite recent advancements in diagnostic and therapeutic modalities, the clinical prognosis for esophageal cancer remains unsatisfactory, with an overall 5-year survival rate of less than 30% (3). Postoperative recurrence and metastasis are the primary causes of treatment failure, with approximately 50% of patients experiencing disease progression following radical resection (4). The current standard treatment regimen involves surgery combined with platinum-based adjuvant chemotherapy; however, chemoresistance is widespread and represents a critical bottleneck hindering efficacy improvement (2, 5). Research indicates that aberrant activation of the DNA damage repair (DDR) pathway can contribute to chemoresistance by enhancing the capacity of tumor cells to repair platinum-induced DNA damage (6, 7). Nonetheless, there is a lack of effective biomarkers to predict patient sensitivity to adjuvant chemotherapy, necessitating an urgent clinical need to develop individualized treatment strategies based on molecular characteristics.

APE1, the core enzyme of the base excision repair (BER) pathway, has garnered significant interest due to its distinctive bifunctional nature (8). This protein exhibits both nucleic acid endonuclease activity, crucial for repairing oxidatively damaged DNA bases, and influences the activity of pivotal transcription factors like NF-κB and STAT3 through redox regulatory mechanisms (9, 10). Previous research indicates that APE1 is abnormally overexpressed in solid tumors, such as gastric cancer and osteosarcoma, and its elevated expression is strongly associated with tumor invasion, metastasis, treatment resistance, and adverse prognosis (11, 12). The nucleocytoplasmic mislocalization of APE1 is also a critical factor in chemoresistance (13). It is particularly noteworthy that the majority of existing studies on APE1 and esophageal cancer have concentrated on EAC, prevalent in Western populations (14, 15). Conversely, there remains a paucity of comprehensive investigations into the expression patterns, regulatory mechanisms, and clinical implications of APE1 in ESCC, the predominant pathological type in China.

In this study, we employed IHC to quantify APE1 protein expression in tumor tissues from 115 patients with ESCC who had undergone radical surgery and standard chemotherapy. The patients were stratified into three groups based on APE1 expression levels using the H-score system: high, intermediate, and low. The primary endpoints of the study were DFS and OS. The association between APE1 expression and prognosis was assessed using Cox proportional hazards regression models and further validated in an independent cohort of 110 ESCC patients using tissue microarrays (TMA) and the GEO dataset GSE5325. Additionally, the relationship between APE1 expression and the tumor immune microenvironment (TIME) was examined through multiplex immunofluorescence (mIF) techniques. The objective of this study was to elucidate the clinical significance of APE1 in ESCC, to develop a prognostic model utilizing molecular characteristics, and to establish a theoretical framework for the advancement of personalized treatment strategies.

## Methods

### Research design and ethics

The study was a single-center, retrospective cohort analysis, which was ethically approved by the Ethics Committee of the Army Medical Center of PLA (Grant No. 2023-189). It adhered to the Declaration of Helsinki and the STROBE reporting guidelines. Informed consent was not required, as the research was based solely on anonymized clinical data and archived pathological samples.

### Inclusion and exclusion criteria

Inclusion criteria were met by consecutive primary patients who underwent radical surgery for ESCC between January 2017 and December 2018. Exclusion criteria comprised: (1) receipt of neoadjuvant therapy prior to surgery, (2) the presence of concomitant malignancies, (3) failure to complete ≥4 cycles of platinum-based chemotherapy postoperatively (Cisplatin 75mg/m² + 5-FU 1000mg/m² every 3 weeks for 4–6 cycles), (4) administration of immunotherapy following surgery, (5) incomplete follow-up data. 115 patients met the inclusion criteria. Their baseline characteristics are presented in **Table 1**.

**Table 1:** Baseline characteristics of ESCC patients.

Clusters	Subtype	APE1			*p*
		Low	Medium	High	
Sex	man	27	37	32	0.48
	woman	8	6	5	
Age	≤65	20	25	25	0.596
	>65	15	18	12	
Smoke	no	13	12	10	0.584
	yes	22	31	27	
Drink	no	12	11	11	0.704
	yes	23	32	26	
Dysphagia	≤2	29	33	31	0.682
	>2	6	10	6	
Grade	Moderate/Well	28	38	31	0.595
	poor	7	5	6	
Lymphovascular invasion	no	25	29	26	0.924
	yes	10	14	11	
Perineural invasion	no	28	29	19	0.036
	yes	7	14	18	
T stage	T0-T2	22	15	10	0.005
	T3-T4	13	28	27	
N stage	N0	21	22	18	0.598
	N1-N3	14	21	19	
Stage	Tis-II	25	29	20	0.265
	III-IV	10	14	17	

### Treatment plan and follow-up

All patients were subjected to radical surgery for esophageal cancer and received platinum-based chemotherapy regimens, such as cisplatin or carboplatin in conjunction with 5-fluorouracil, paclitaxel, and others, postoperatively. Follow-up commenced immediately following the first postoperative treatment and concluded on December 31, 2024. The duration of follow-up was up to five years, with disease progression or death from any cause designated as the endpoint, and non-progressors or survivors were censored at their most recent follow-up visit. Concurrently, we retrieved the GSE53625 dataset from the Gene Expression Omnibus (GEO) database (https://www.ncbi.nlm.nih.gov/gds/). After rigorous filtering, this dataset comprised 149 ESCC patients, including 104 who received adjuvant therapy and 45 who did not.

### Hematoxylin and eosin staining

The tissues were embedded in paraffin, dewaxed with xylene, rehydrated in an ethanol series (100%, 90%, 80%, 70%), and immersed in water. Then, the sections were stained with hematoxylin for 3–5 minutes, washed in tap water and differentiated with 1% acid alcohol for a few seconds to red before being rinsed in running water. Next, the sections were stained in 1% eosin for 10 minutes and washed with tap water. Finally, the sections were dehydrated by increasing concentrations of alcohol and mounted with coverslips.

### Immunohistochemistry

The paraffin-embedded tissues sections were deparaffinized using xylene and then rehydrated through an ethanol series. For antigen retrieval, the slides were autoclaved in 10 mM sodium citrate buffer. Next, the sections were pre-incubated twice in PBS and incubated overnight at 4°C with the following primary antibody dilutions: APE1 (ab137708, Abcam), at a dilution of 1:400. The sections were then rinsed twice with PBS and incubated with its associated HRP-conjugated secondary antibody for 30 minutes at room temperature. After that, the sections were further rinsed with PBS and diaminobenzidine (DAB) was added dropwise. When a clear brownish yellow positive signal appeared, the slide was placed in flowing water to stop the colorimetric process. Finally, the sections were placed in hematoxylin for nuclear staining. In the experiment, skin tissues from wild-type mice and systemic APE1 knockout mice served as the positive and negative controls, respectively.

### Multiplex immunohistochemistry

Pathology slides were incubated in an oven at 65°C for 3 hours to facilitate dehydration. Subsequent deparaffinization involved immersion in xylene for 10 minutes, repeated three times. Following fixation in a 4% paraformaldehyde solution for 20 minutes, acid/base repair was conducted for the targeted antigen. After a 10-minute sealing period, primary antibodies were introduced and stained. The repair, primary antibody incubation, and staining steps were then repeated. The specific antibodies and dyes employed during the experiment were APE1 (Abcam, 137708), CD8 (Abcam, 316778), FOXP3 (CST <ns/>87048), CD163 (Abcam, 283654), a-SMA (CST <ns/>19245), FAP (CST <ns/>52818), CD4 (Abcam, 1336116). The treated slices were subsequently blocked and mounted on the AkoyaOpal assay platform. Immunofluorescence intensity was statistically analyzed by imageJ.

### Model development

The prediction model was constructed using the XGBoost algorithm, an ensemble machine learning method that integrates decision trees with a gradient boosting framework and is particularly suitable for survival analysis modeling of high-dimensional biomedical data. Employing the Cox proportional hazards model as the optimization objective (objective = "survival:cox"), the model minimizes the negative log-likelihood loss function (eval_metric = "cox-nloglik") to analyze survival data, with the framework inherently supporting handling of censored data and enhancing predictive accuracy through iterative training of multiple weak decision trees. Core hyperparameters were configured as follows: learning rate (η) = 0.05 to balance convergence speed and generalization capability; maximum tree depth (max_depth) = 3 to constrain individual tree complexity; L1 regularization (alpha) = 0.5 and L2 regularization (lambda) = 2 to dually control model complexity while promoting feature sparsity; column sampling rate (colsample_bytree) = 0.5 enabling random selection of 50% features per tree for enhanced robustness; minimum child weight (min_child_weight) = 3 to restrict leaf-node sample weight thresholds and mitigate noise impacts. Model stability was evaluated via 4-fold stratified cross-validation with a fixed random seed (19891008) ensuring reproducibility, while training employed a maximum of 500 iterations (nrounds) with early stopping (early_stopping_rounds = 20) triggered when the validation-set Cox negative log-likelihood showed no improvement for 20 consecutive rounds.

### SHAP analysis

To interpret the model and identify the most critical predictors influencing ESCC patient prognosis, SHAP (SHapley Additive exPlanations) values were computed using the shapviz package in R. SHAP values quantify the contribution of each feature to individual predictions. The analysis identified two pivotal prognostic factors for ESCC patients, with results visualized through SHAP summary plots to illustrate the directional impact and relative importance of each variable.

### Statistical analysis

SPSS version 25.0 was employed for the analysis of data. Count data were presented as frequencies (percentages), and the χ² test or Fisher’s exact probability test was utilized for group comparisons. Survival analysis was conducted using the Kaplan-Meier method, and the Log-rank test was applied to assess group differences. Prognostic factors were investigated via univariate and multivariate Cox proportional hazard models, which were used to identify independent prognostic indicators. All statistical tests were conducted at a two-sided significance level, with a P-value of <0.05 deemed statistically significant.

## Results

### Baseline characteristics of patients

**Figure 1:**
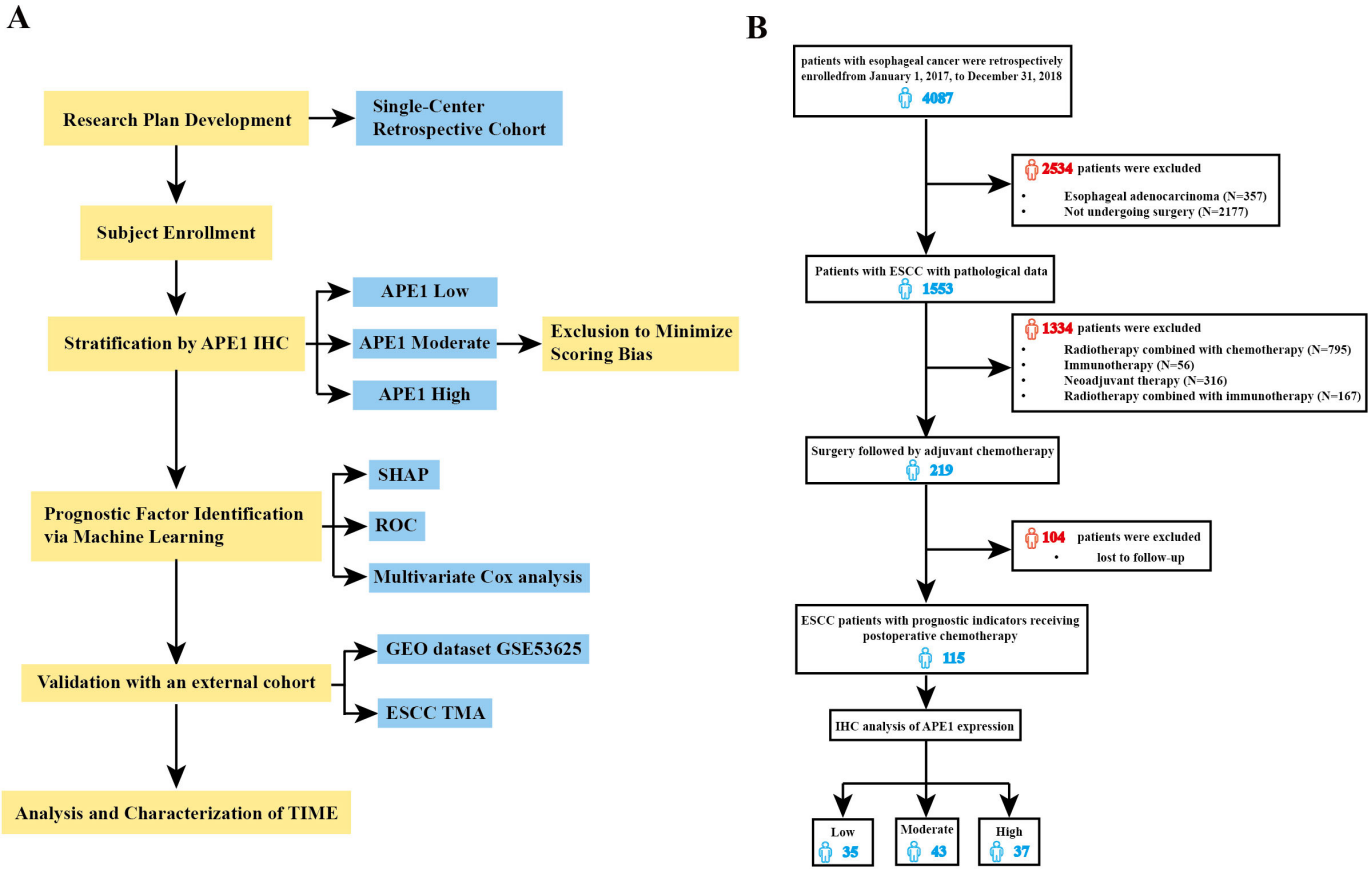
Flow diagram of selection of eligible patients. **(A)** Study flowchart. **(B)** Conforming to the established inclusion and exclusion criteria, 115 patients were stratified into three distinct groups based on the expression levels of APE1: low (n=35), medium (n=43), and high (n=37) expression.

To further investigate the clinical value and utility of APE1 as a prognostic biomarker in ESCC, we performed this study according to the research workflow detailed in **Figure 1A**. A total of 115 patients with ESCC who received chemotherapy after surgery were included in this study. The inclusion and exclusion criteria are shown in **Figure 1B**, and baseline characteristics are presented in **Table 1**. This retrospective study analyzed data from 4,087 esophageal cancer patients treated at our hospital between 2017 and 2018. Of these, 1,533 underwent surgical resection at our institution. After applying inclusion criteria—availability of archived surgical paraffin-embedded specimens and receipt of only platinum-based adjuvant chemotherapy—219 patients were enrolled. These individuals were followed for five years. As of December 31, 2024, complete five-year survival data were available for 115 patients, who formed the final study cohort. The study protocol was approved by the Ethics Committee of the Army Medical Center of the PLA (Approval No. 2023-189). Tumor tissue samples were obtained from Specimen No. 1 (i.e., representative tumor tissue) archived in the hospital’s Department of Pathology. Overall Survival (OS) was defined as the primary endpoint, and Disease-Free Survival (DFS) as the secondary endpoint.

The cohort comprised 96 males (83.5%) and 19 females (16.5%). Age distribution was as follows: 45 patients (39.1%) were > 65 years, and 70 patients (60.9%) were ≤ 65 years. According to disease stage, 74 patients (64.4%) were classified as early-stage (Tis-II stage), while 41 patients (35.6%) had advanced disease (Stage III-IV).

### Association of APE1 expression with clinical and pathological characteristics

**Figure 2:**
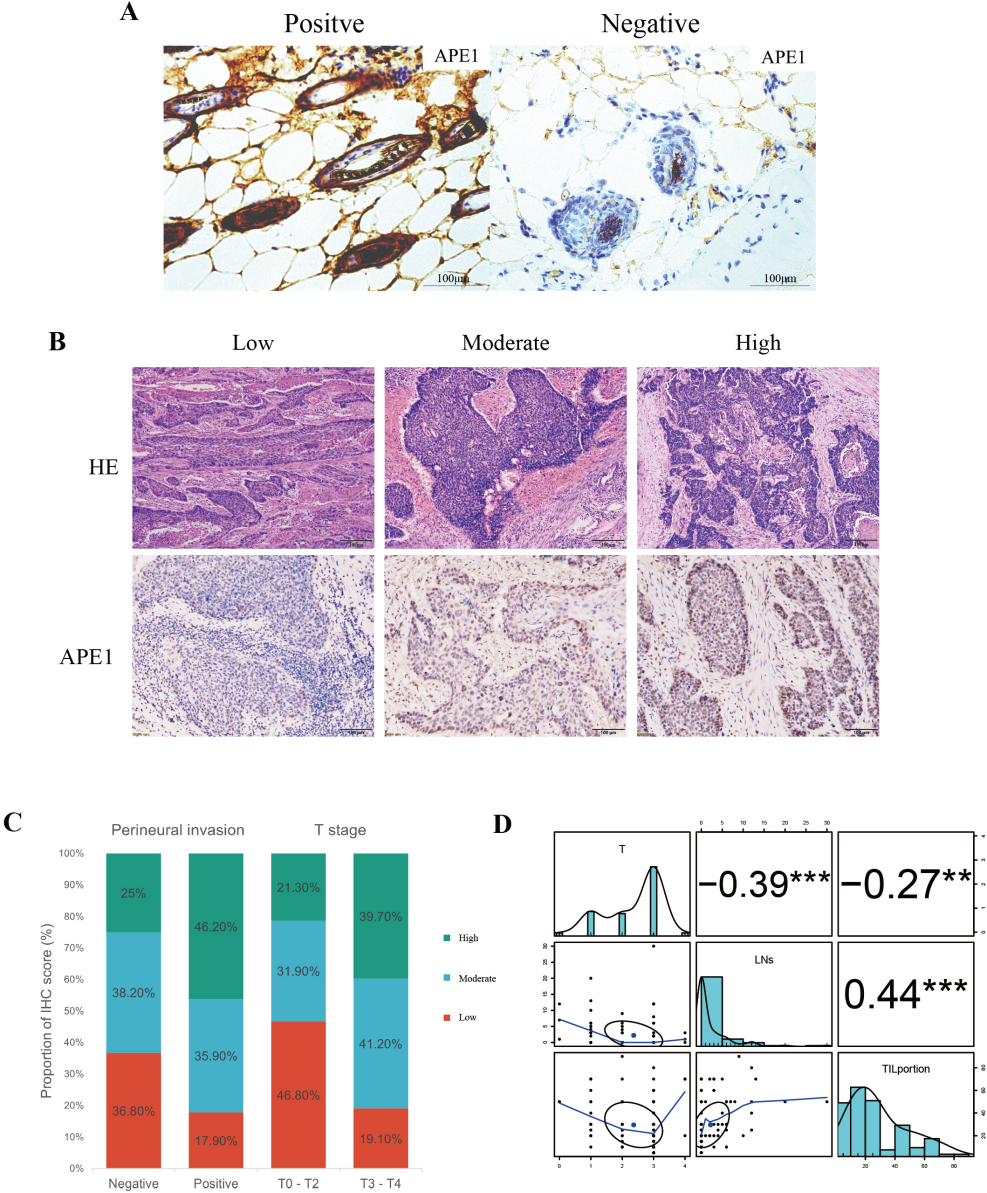
Correlation between APE1 expression and clinicopathological features. **(A)** Positive and Negative Controls for IHC. **(B)** HE staining and IHC staining of APE1of patient tumor specimens. **(C)** Percentage bar charts of patients in different subgroups in perineural invasion and T stage. **(D)** Analysis of patient T-staging in correlation with TIL and TLS, “**” indicates p < 0.01 and “***” indicates p < 0.001.

The specificity of APE1 immunostaining was definitively characterized using a tamoxifen-inducible whole-body APE1 knockout model (10). Wild-type mouse tissues showed positive staining, confirming antibody efficacy, whereas knockout mouse tissues showed a complete absence of staining, establishing a definitive negative control (**Figure 2A**). Tissue sections from 115 patients were independently assessed by two pathologists blinded to the experimental groups using a semi-quantitative scoring system (-: no staining; +: weak staining; ++: moderate staining; +++: strong staining). The inter-observer agreement was assessed using Cohen's Kappa statistic, which yielded a value of 0.72. According to the benchmark set by Landis and Koch, this value represents substantial agreement, indicating that our scoring criteria are clear and the results are reliable (16).

APE1 expression levels were categorized into three groups: low (-/+, n=35, 30.4%), moderate (++, n=43, 37.4%), and high (+++, n=37, 32.2%) (**Figure 2B**). With the exception of T-stage (*p* = 0.005) and perineural invasion (*p* = 0.036), no significant differences were observed in baseline characteristics among other patients (**Table 1**). To minimize potential bias introduced by the aforementioned scoring method, we dichotomized the patient cohort into APE1 high and low expression groups for comparison. Correlation analysis revealed that high APE1 expression was significantly associated with perineural invasion (46.2% vs. 17.9%, *p* = 0.036) and advanced T-stage (T3-T4: 39.7% vs. T1-T2: 19.1%, *p* = 0.005) (**Figure 2C**). No significant associations were observed with gender, age, smoking status, alcohol consumption, histopathological grade, or lymphovascular invasion. These findings suggest that patients with elevated APE1 expression may exhibit more extensive tumor infiltration. Quantification of tumor-infiltrating lymphocytes (TIL) and tertiary lymphoid structures (TLS) was performed via HE staining (**Figure 2B**), followed by Spearman correlation analysis with T-stage. Results demonstrated significant negative correlations between T-stage and both TIL density (R = -0.27, P = 0.004) and TLS density (R = -0.39, *p* < 0.001) (**Figure 2D**, **Supplementary Figures 1A, B**). Notably, a strong positive correlation was observed between TIL and TLS levels (R = 0.44, *p* < 0.001) (**Figure 2D**). Furthermore, non-smokers or patients with early T-stage exhibited significantly higher TLS and TIL counts compared to smokers or those with advanced T-stage (**Supplementary Figures 1C-1F**).

### Impact of APE1 expression on survival prognosis

**Figure 3:**
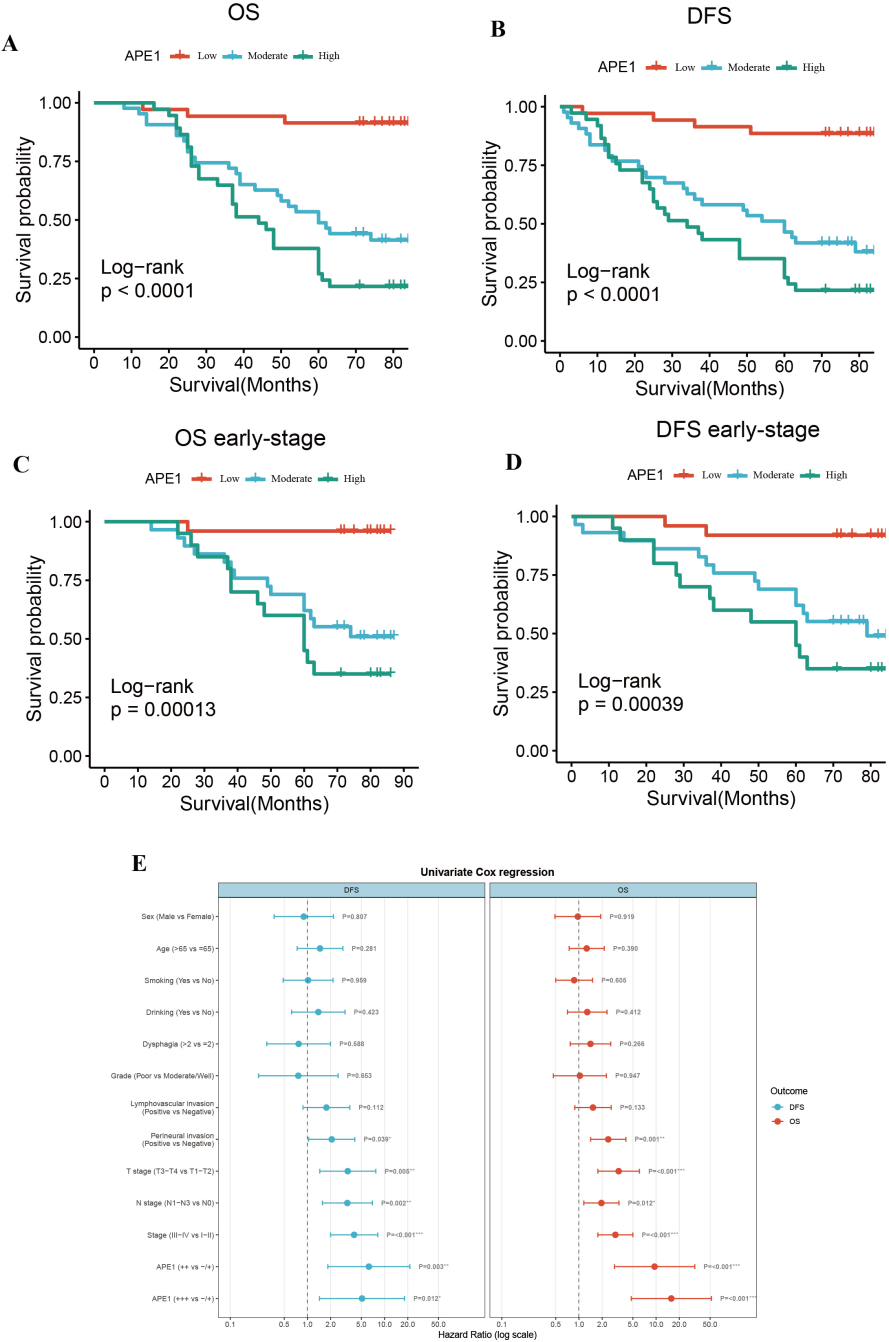
Overall survival and disease-free survival. **(A)** OS of the entire cohort. **(B)** DFS of the entire cohort. **(C)** OS of patients with early stage ESCC. **(D)** DFS of patients with early stage ESCC. **(E)** Forest plot of risk ratios associated with the prognosis of postoperative chemotherapy combinations for ESCC.

To mitigate the limitations associated with semi-quantitative assessment, we dichotomized the patient cohort into discrete groups with high or low APE1 expression for a robust investigation of its role in ESCC. Kaplan-Meier analysis demonstrated that the low APE1 expression patients had significantly longer median OS compared to the high-expression (79 months vs. 44 months; HR = 15.74, 95% CI 4.75–52.15, *p* < 0.001), and similarly exhibited superior median DFS (77 months vs. 34 months; HR = 11.85, 95% CI 4.13–34.00, *p* < 0.001) (**Figures 3A, B**). Subgroup analysis of early-stage operable patients (Tis-II stage) further revealed that high APE1 expression remained significantly associated with poorer outcomes: the low-expression group showed a median OS of 80 months versus 60 months in the high-expression group (HR = 23.52, 95% CI 3.06–180.81, *p* < 0.001), and a median DFS of 80 months versus 60 months (HR = 11.58, 95% CI 2.60–51.59, *p* < 0.001) (**Figures 3C, D**). Univariate Cox regression analysis identified perineural invasion, T stage, N stage, clinical stage, and APE1 expression as significant predictors for both DFS and OS (**Figure 3E**, **Table 2**).

**Table 2:** Univariate Cox analyses of OS and DFS for ESCC in the cohort.

Univariate Cox regression	DFS		OS	
	HR (95% CI)	*p*	HR (95% CI)	*p*
Sex (Male vs Female)	0.895 (0.370-2.169)	0.807	0.965 (0.488-1.910)	0.919
Age (>65 vs ≤65)	1.456 (0.735-2.882)	0.281	1.258 (0.746-2.124)	0.390
Smoking (Yes vs No)	1.020 (0.485-2.143)	0.959	0.865 (0.498-1.500)	0.605
Drinking (Yes vs No)	1.385 (0.625-3.072)	0.423	1.280 (0.710-2.308)	0.412
Dysphagia (>2 vs ≤2)	0.769 (0.297-1.991)	0.588	1.409 (0.770-2.576)	0.266
Grade (Poor vs Moderate/Well)	0.761 (0.232-2.496)	0.653	1.027 (0.465-2.266)	0.947
lymphovascular invasion (Positive vs Negative)	1.762 (0.876-3.545)	0.112	1.517 (0.880-2.615)	0.133
Perineural invasion (Positive vs Negative)	2.067 (1.038-4.114)	0.039	2.395 (1.416-4.049)	0.001
T stage (T3-T4 vs T1-T2)	3.330 (1.443-7.687)	0.005	3.268 (1.756-6.081)	0.000
N stage (N1-N3 vs N0)	3.291 (1.563-6.931)	0.002	1.953 (1.155-3.301)	0.012
Clinical Stage (III-IV vs I-II)	4.029 (1.993-8.146)	0.000	2.963 (1.755-5.003)	0.000
APE1 (Moderate vs Low)	6.256 (1.840-21.269)	0.003	9.548 (2.879-31.662)	0.000
APE1 (High vs Low)	5.104 (1.434-18.166)	0.012	15.734 (4.766-51.938)	0.000

### SHAP analysis further revealed APE1 as an independent prognostic factor

**Figure 4:**
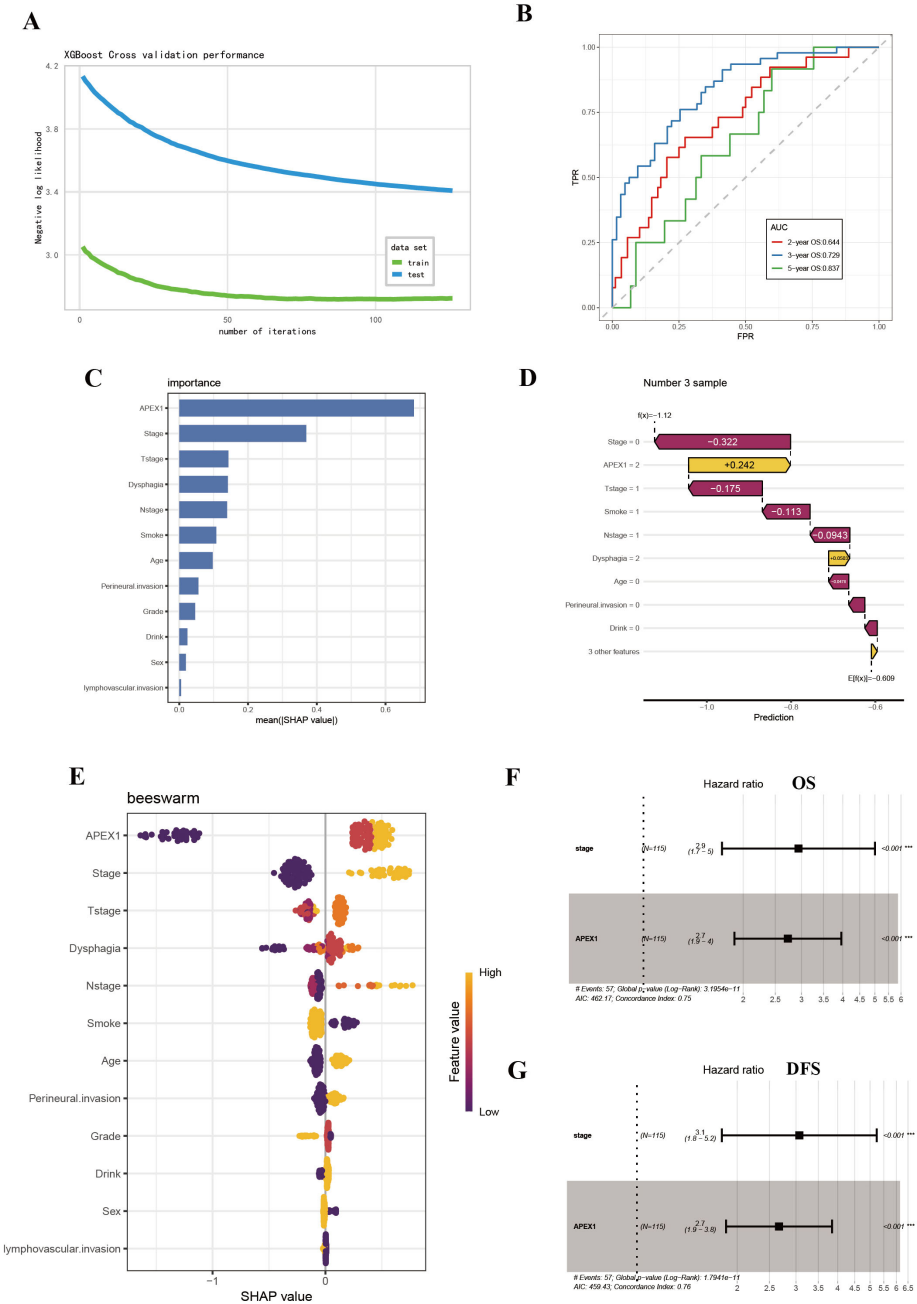
SHAP analysis further revealed APE1 as a significant prognostic factor. **(A)** Log-Likelihood Curve of XGBOOST model. **(B)** AUC curve of XGBoost model. **(C)** SHAP summary plot. **(D)** Visualization of SHAP feature importance ranking correlation. **(E)** SHAP waterfall plot. **(F, G)** Multivariate Cox regression analysis for prognosis in ESCC patients.

Based on the univariate Cox regression analysis, we further employed the eXtreme Gradient Boosting (XGBoost) machine learning algorithm combined with SHAP (SHapley Additive exPlanations) value analysis to rank feature importance, with the aim of identifying predictors that have a significant impact on prognosis. The XGBoost model demonstrated robust prognostic discrimination in 4-fold cross-validation: Cox negative log-likelihood was 3.439 ± 0.033 for the training set and 2.719 ± 0.129 for the test set, with optimal iteration achieved at 106 rounds. Time-dependent ROC curve analysis revealed the following predictive performance on the internal validation set: 2-year AUC = 0.644, 3-year AUC = 0.729, and 5-year AUC = 0.837, indicating enhanced predictive capability for long-term survival outcomes (**Figures 4A, B**).

The SHAP summary plot visualized the global impact of features on model predictions (**Figure 4C**), while SHAP feature importance ranking identified APE1 expression level and clinical stage as key prognostic determinants based on mean absolute SHAP values (**Figure 4D**). Individual prediction interpretation via SHAP waterfall plots revealed that: red features decreased SHAP values (indicating poorer prognosis), yellow features increased SHAP values (suggesting favorable prognosis), with color bar length representing contribution magnitude. The baseline prediction value is denoted as E[f(x)] (**Figure 4E**). Shapley value-based interpretability analysis revealed that tumor stage and APE1 expression level play dominant roles in influencing patient prognosis, with their contributions far exceeding those of other variables. Subsequent multivariate COX analysis demonstrated that both tumor APE1 expression (OS: HR = 2.7, 95% CI 1.9–4.0, *p* < 0.001; DFS: HR = 2.7, 95% CI 1.9 - 3.8, *p* = < 0.001 ) and tumor stage (OS: HR = 2.9, 95% CI 1.7–5.0, *p* < 0.001; DFS: HR = 3.1, 95% CI 1.8–5.2, *p* < 0.001 ) were independent prognostic factors for both OS and DFS (**Figures 4F, G**).

### Independent validation of the prognostic significance of APE1 in ESCC

**Figure 5:**
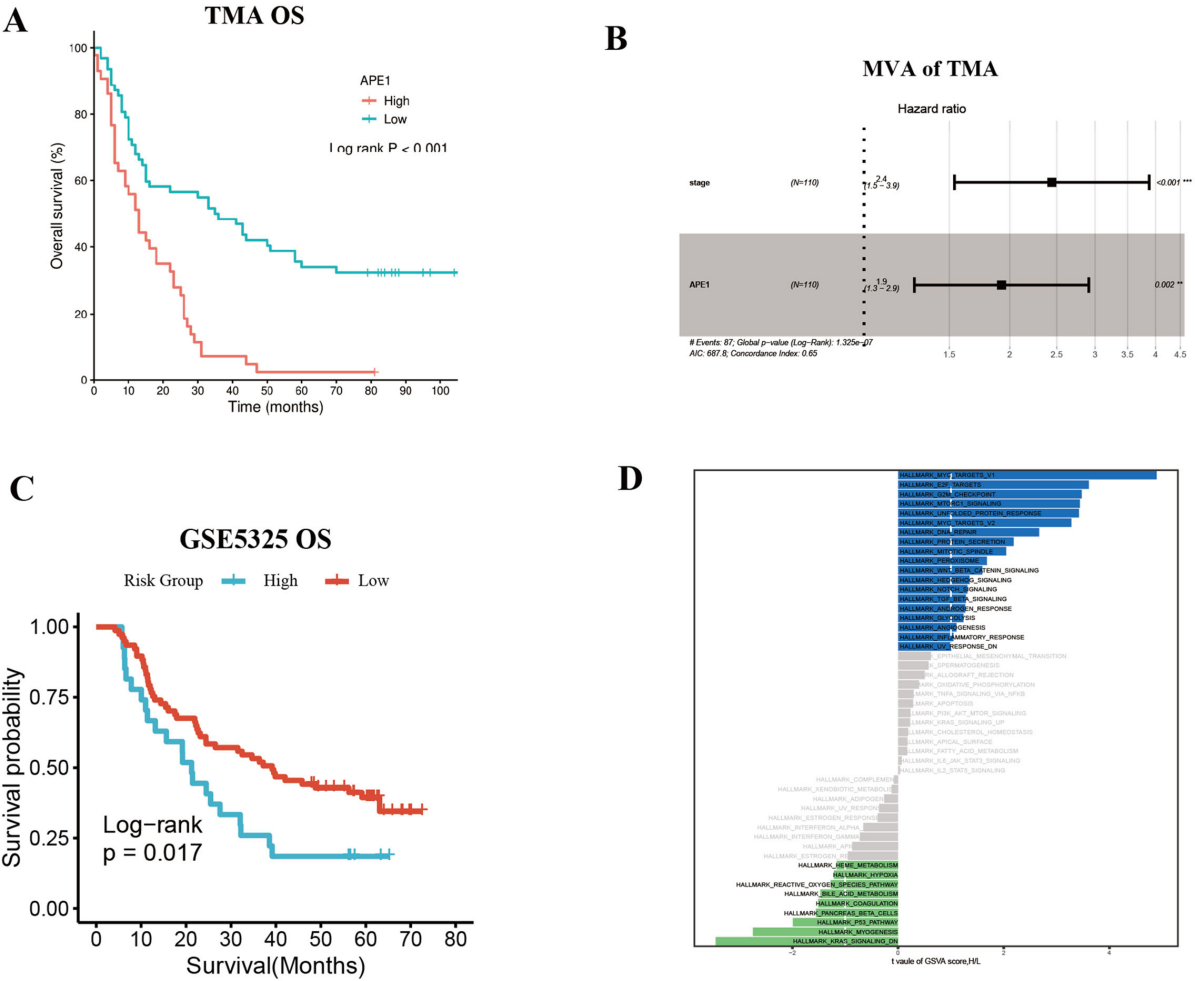
Independent validation of the prognostic significance of APE1 in ESCC. **(A)** OS of TMA patients. **(B)** Multivariate Cox regression analysis for TMA patients. **(C)** Survival analysis in ESCC patients with adjuvant Chemotherapy in dataset GSEA53625. **(D)** GSVA enrichment analysis result plot.

To further substantiate these findings, we utilized the Tissue Microarray (TMA) (ID: HEsoS180Su10; Shanghai Outdo Biotech Co., Ltd.) as an independent validation cohort. This TMA consists of tumor tissues from 110 ESCC patients who received postoperative chemotherapy. The baseline clinical characteristics of these patients are summarized in **Table 3**. Prognostic analysis revealed that patients with high APE1 expression had a significantly shorter overall survival than those with low expression (mOS: 12m vs. 35m months; HR: 2.53; 95% CI: 1.63 - 3.93; p < 0.001), consistent with the results from our initial clinical cohort (**Figure 5A**). Furthermore, multivariable analysis identified both APE1 expression level (HR = 1.9; 95% CI = 1.3 - 2.9; p = 0.002) and clinical stage (HR = 2.4; 95% CI = 1.5 - 3.9; p < 0.001) as independent prognostic factors for ESCC in this TMA cohort (**Figure 5B**). In conclusion, this external validation using an independent, non-center-specific patient cohort confirms that APE1 expression is a critical determinant of prognosis in ESCC.

**Table 3:** Baseline characteristics of TMA ESCC patients.

Group	Subtype	APE1		*p*
		L	H	
Sex	Men	40	41	0.007
	Women	23	6	
Age	≤65	30	22	0.8
	>65	33	25	
T stage	T1-T2	15	9	0.386
	T3-T4	48	38	
N stage	N0	33	17	0.079
	N1-N3	30	29	
Stage	I-II	37	16	0.007
	III	26	31	

Additionally, we corroborated these findings using publicly available datasets from the GEO database, using the GSE5325 dataset we conducted an analysis of APE1 expression levels and prognosis in 179 ESCC patients. Patient baseline characteristics are detailed in **Supplementary Table 1**. Among patients who received postoperative adjuvant chemotherapy, those with high APE1 expression exhibited a significantly lower OS compared to the low expression group (HR = 1.85, 95% CI = 1.11–3.08, *p* = 0.017) (**Figure 5C**). GSVA score results revealed that compared to patients with low APE1 expression, those with high APE1 expression not only exhibited enhanced DNA repair activity, but also showed significant upregulation of the WNT/β-catenin signaling, TGFβ signaling, and inflammatory response (**Figure 5D**). The activation of these pathways has been established in multiple cancers to be associated with an unfavorable prognosis (17–19).

### Tumor immune microenvironment analysis

**Figure 6:**
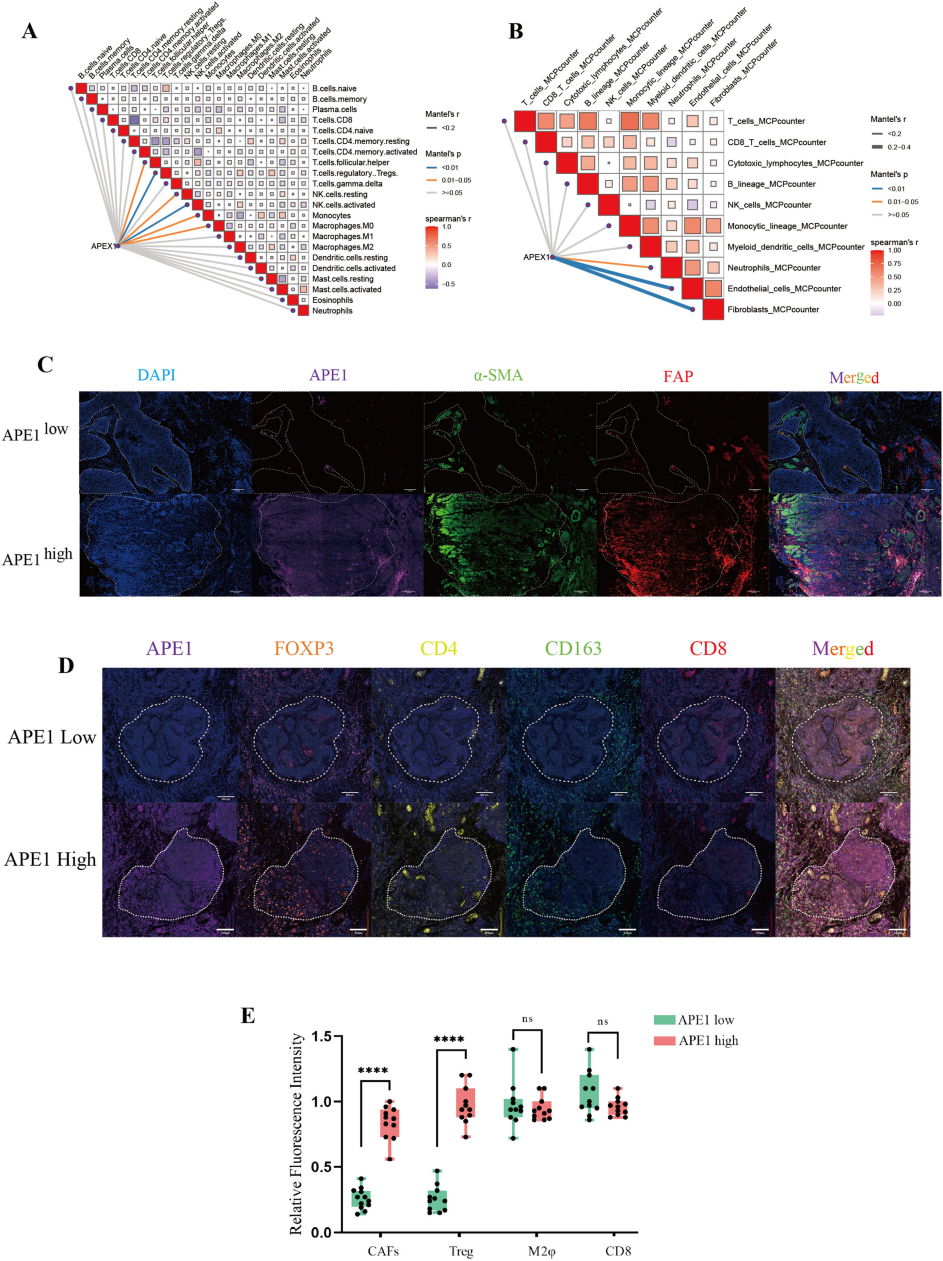
Immunological microenvironmental analysis. **(A, B)** GEO database GSE53625, network diagram of APEX1 correlation with immune infiltration analysis. **(C)** mIHC detection of APE1, a-SMA and FAP expressions in patient pathology specimens, scale bar = 100 μm. **(D)** mIHC detection of FOXP3, CD163, CD4 and CD8 expressions in patient pathology specimens mice, scale bar = 200 μm. **(E)** Statistical analysis of the relative fluorescence intensities of a-SMA, FAP, FOXP3, CD4, CD163 and CD8 in different patients (n = 10), “****” indicates *p* < 0.0001 and “ns” indicates none significance.

During HE staining of patient samples, we observed that the density of fibroblasts surrounding tumors was significantly higher in patients with high APE1 expression compared to those with low APE1 expression (**Figure 2B**). Furthermore, we performed a correlation analysis between APE1 expression levels and the infiltration levels of immune cells within the TIME in the aforementioned dataset. The results demonstrated that APE1 expression exhibited significantly positive correlations with the activation of Tregs, natural killer (NK) cells activation, cancer-associated fibroblasts (CAFs), and endothelial cells (**Figures 6A, B**).

To further investigate this phenomenon, we performed mIF analysis on CAFs isolated from both APE1-high and APE1-low expression groups. We adopted a representative sampling strategy by selecting 10 patients per group that best reflected key clinicopathological characteristics. The results demonstrated that the expression levels of α-SMA and FAP were significantly elevated from the APE1-high group relative to the APE1-low group (**Figures 6C, E**) (20). Furthermore, analysis of key immune cell populations within the TIME revealed that the density of FOXP3^+^ and CD4^+^ Treg cells was significantly higher in the APE1-high group (21). In contrast, the densities of CD163^+^ macrophages and CD8^+^ T cells showed no statistically significant differences between the two groups (**Figures 6D, E**) (22).

## Conclusion

In patients with ESCC who underwent postoperative adjuvant chemotherapy, Elevated APE1 expression is significantly associated with advanced T-stage, resulting in markedly reduced densities of TIL and TLS in the peritumoral region. Furthermore, high expression of APE1 in tumor tissues was significantly associated with poor prognosis and could serve as a novel independent prognostic biomarker distinct from clinical staging. The underlying mechanism involve increased infiltration of FOXP3^+^ and CD4^+^ Treg cells and CAFs within the tumor microenvironment. The APE1-centered hypothesis presented herein provides a framework for treatment personalization: single-agent chemotherapy suffices for low-APE1 ESCC, whereas high-APE1 cases warrant combinatorial targeting of both tumor invasion and immune evasion pathways (Graphic Abstract). This study provides a novel biomarker for personalized treatment of ESCC, facilitating the identification of patient subgroups that may benefit from immunochemotherapy and optimizing postoperative adjuvant therapeutic strategies.

## Discussion

This study presents a systematic analysis of the clinicopathological features and molecular expression profiles in 115 cases of operable ESCC. It represents the comprehensive demonstration that elevated APE1 expression is significantly correlated with adverse prognostic outcomes in patients receiving postoperative adjuvant chemotherapy. Multivariate Cox regression modeling confirmed that high APE1 expression serves as an independent prognostic indicator for both DFS and OS. This statistical significance remained robust even after adjusting for key clinicopathological parameters, including T-stage, perineural invasion, and other relevant factors (**Figure 4**, **Table 2**). Of particular note, within the subgroup of T1–2 stage patients, high APE1 expression effectively stratified distinct prognostic trajectories. In clinical practice, the majority of patients eligible for surgery present with early-stage disease. Our findings indicate that APE1 expression also serves as a strong prognostic biomarker for chemotherapy response in this patient population. This provides a novel biomarker that may aid in predicting the effectiveness of chemotherapy in clinical settings. These findings are also consistent with previous investigations into DNA repair proteins in therapeutic resistance but introduce an innovative focus on the adjuvant therapy context, positing APE1 as a novel biomarker for individualized treatment stratification (23, 24).

From a mechanistic perspective, mIF analyses revealed that APE1-high tumors exhibited augmented infiltration of FOXP3^+^ regulatory T cells and a positive correlation with α-SMA expression, a marker of CAFs (25). Moreover, within the context of immunotherapy, both Treg cells and CAFs are known contributors to the formation of a suppressive immune microenvironment (26, 27). This observation suggests that APE1 expression levels may also correlate with the prognosis of immunotherapy. While this study did not delve into the mechanistic underpinnings of how APE1 contributes to the observed phenomena, future investigations will focus on elucidating the underlying mechanisms. This immunosuppressive tumor microenvironment is hypothesized to arise from APE1-mediated activation of the TGF-β/Smad signaling pathway (28). Notably, whereas chemotherapy-induced damage-associated molecular patterns (DAMPs) typically elicit pro-tumor immune responses, APE1-driven Treg recruitment likely counteracts this effect, providing a plausible mechanistic explanation for observed chemoresistance. Additionally, CAF-Treg crosstalk may establish physical barriers and secrete immunosuppressive cytokines such as IL-10, collectively impeding drug penetration and fostering the development of multi-layered resistance mechanisms (29).

Collectively, we propose the following scientific hypothesis: In ESCC patients undergoing radical resection with adjuvant chemotherapy, elevated APE1 expression (1) promotes increased tumor infiltration depth and advanced T-stage, and (2) induces significant enrichment of CAFs and Tregs, fostering an immunosuppressive tumor microenvironment that ultimately contributes to poor chemotherapeutic outcomes. This implies that early-stage ESCC patients with low APE1 expression may derive greater benefit from postoperative single-agent chemotherapy, whereas those with high expression may require combinatorial therapeutic strategies.

The study also has some limitations including its single-center retrospective design, which introduces potential selection bias, and a moderately sized sample constrained by the requirements of long-term follow-up. The stratification of APE1 expression into three groups served as a *post hoc*, exploratory measure in our study. It was not based on pre-established thresholds nor subjected to statistical optimization, and we contextualize its associated limitations here. The statistical power of the multivariate model in this cohort may be constrained by the number of events (37/115), which stems from our focus on a radically resected esophageal cancer population that exhibits a more favorable prognosis and a lower baseline event rate. Although retrospective studies inherently face challenges in establishing causality, our approach utilizing direct analysis of surgical specimens to predict postoperative response to chemotherapy may help mitigate this limitation. Future multi-center prospective investigations are essential to validate the clinical utility of APE1 and further elucidate its precise immunomodulatory mechanisms within the tumor microenvironment.

## Data Availability

The raw data supporting the conclusions of this article will be made available by the authors, without undue reservation.

## Supplementary material

Supplementary Figure 1 Correlation between APE1 expression and clinicopathological features. **(A)** T-stage and TLS correlation analysis. **(B)** T-stage and TIL correlation analysis. **(C)** T-stage and TLS correlation analysis. **(D)** T-stage and TIL correlation analysis. **(E)** Smoking and TLS correlation analysis. **(F)** Smoking and TIL correlation analysis.
