# Supplementary figures and images for: APE1 mediates chemoresistance in esophageal squamous cell carcinoma by remodeling the immunosuppressive microenvironment
Source: Front Immunol. 2025 Oct 29;16:1689468. doi: 10.3389/fimmu.2025.1689468 (PMC12605184; doi:10.3389/fimmu.2025.1689468)

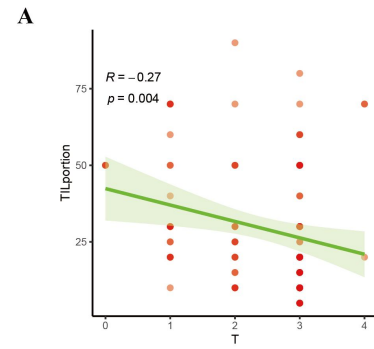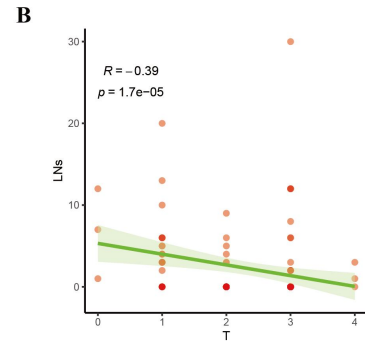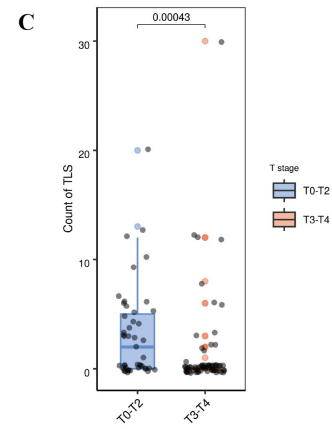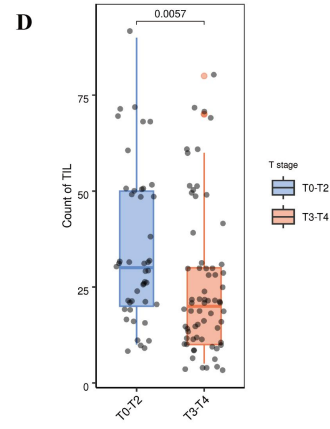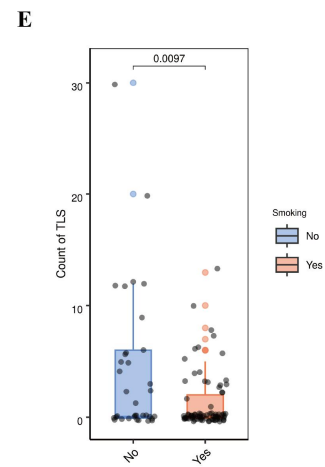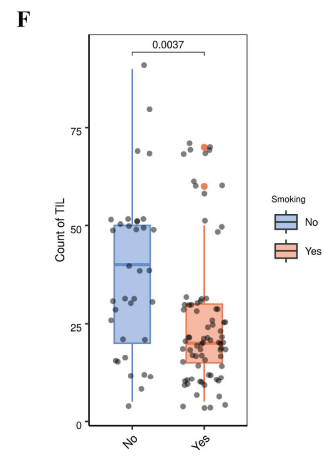

Supplement: Supplementary Figure 1 — Correlation between APE1 expression and clinicopathological features. (A) T-stage and TLS correlation analysis. (B) T-stage and TIL correlation analysis. (C) T-stage and TLS correlation analysis. (D) T-stage and TIL correlation analysis. (E) Smoking and TLS correlation analysis. (F) Smoking and TIL correlation analysis. [file Image1.pdf]
